# Predictors for Anterior Cruciate Ligament (ACL) Re-injury after Successful Primary ACL Reconstruction (ACLR)

**DOI:** 10.5704/MOJ.2011.009

**Published:** 2020-11

**Authors:** R Gupta, A Singhal, A Malhotra, A Soni, GD Masih, M Raghav

**Affiliations:** Department of Orthopaedics, Government Medical College Hospital Chandigarh, Chandigarh, India

**Keywords:** ACL re-injury, knee, athletes, return to sports

## Abstract

**Introduction:**

Few authors have addressed risk factors related to an ipsilateral graft rupture and contralateral anterior cruciate ligament (ACL) injury after return to sports (RTS) following primary ACL reconstruction.

**Material and Methods:**

Patients with ACL re-injury to either knee after successful primary ACLR were included in Group I and those with no further re-injury were included in Group II. Variables including age, gender, side, body mass index (BMI), thigh atrophy, anterior knee laxity difference between both knees measured by KT-1000 arthrometer, mean time of return to sports (RTS), graft type, type of game, mode of injury, Tegner Activity Score, hormone levels, femoral tunnel length (FTL), posterior tibial slope (PTS) and notch width index (NWI) were studied. Binary logistic regression was used to measure the relative association.

**Results:**

A total of 128 athletes were included with 64 in each group. Mean age in Group I and II were 24.90 and 26.47 years respectively. Mean follow-up of Group I and Group II were 24.5 and 20.11 months respectively. Significant correlation was present between ACL re-injury and following risk factors; PTS of >10º, KT difference of >3.0mm, thigh atrophy of >2.50cm and time to RTS <9.50 months P value <0.05). No correlation was found with age, sex, BMI, type of game, Tegner Activity Score, mode of injury, NWI, size of graft, FTL and hormone levels.

**Conclusion:**

Possible risk factors include PTS of ≥ 10º, KT difference of ≥ 3.0mm at 1 year follow-up, thigh atrophy of ≥ 2.50cm at 1 year follow-up and RTS <9.5 months after primary ACLR.

## Introduction

The anterior cruciate ligament (ACL) is the major passive restriction to anterior translation of the tibia on the femur, with the reported incidence of primary ACL injury as 1.5% to 1.7% per year in healthy athletic population^1,2^. After an ACL injury, athletes present with the main complaint of knee instability^3^, for which ACL reconstruction (ACLR) is the current gold standard operative management^4^. A recent patient satisfaction survey concluded that athletes who can resume their sporting activity are more likely to be satisfied with the outcome of the ACLR^5^.

Several risk factors for primary ACL injury have been studied and identified. Female sex^6^, race^7^ and participation in pivoting sports^8^ have been widely reported as risk factors for primary ACL tear. Other reported risk factors include enhanced posterior tibial slope^9^, narrow notch width^10^, small size ACL^11^, limb malalignment^11^, neuromuscular control^11^, vertical directed and short femoral tunnel length^12^, and graft tunnel length^13^.

In recent years, there has been tremendous improvement in surgical techniques, methods of fixation and rehabilitation protocol relating to ACL reconstruction^14^. Despite this, the reported incidence of ACL re-injury remains high; 6% for ipsilateral graft injury and another 6% for contralateral knee ACL tear^15^.

In addition, an earlier study^16^ has reported that the ACL graft rupture is a re-injury rather than the graft failure, which further emphasises on the importance of knowing the risk factors which predispose an athlete for ACL re-injury. Only a few authors have addressed risk factors related to an ipsilateral graft rupture and contralateral ACL injury after return to sports (RTS) following primary ACLR^17,18^. Literature is still controversial over the argument of ACL reinjury risk factors to either knee after primary ACL reconstruction in athletes. Keeping racial factor as constant, this study was performed to find risk factors for ipsilateral graft rupture and contralateral ACL injury in athletes.

## Materials and Methods

In this study, patients who had undergone primary ACL reconstruction in the last 10 years were included.

Group I comprised of patients that had ACL re-injury to either knee post primary ACL reconstruction and Group II comprised of patients that had no further ACL injury post primary ACL reconstruction. Inclusion criteria were age 20 to 40 years, both sexes, injury during sports activity, ACLR using autografts semitendinosus gracilis free (STGF), semitendinosus gracilis with preserved insertion (STGPI) or bone patellar tendon bone (BPTB), clinical and magnetic resonance imaging (MRI) evidence of ACL deficient knee. Exclusion criteria were multiple knee ligament injuries, mode of injury other than sports, previous history of surgery on the knee other than ACLR, skeletally immature, infective or inflammatory pathology in the same knee previously or currently and patients who were not willing to participate in study.

The data for the study variables was taken from the pre-filled proformas and inpatient records maintained in our department and medical records department respectively. Both the groups were compared for potential risk factors including age, gender distribution, mode of injury (contact or non-contact), type of sport played, graft type, graft diameter, time to return to sports post primary ACL reconstruction, body mass index, thigh muscle atrophy, arthrometric (KT difference) side to side translation, Tegner Activity Score, posterior tibial slope, notch width index, eostrogen and progesterone levels.

The surgical procedure was performed by the single surgeon (R.G.) in all cases with the same standard technique as of STGPI (semitendinosus gracilis with preserved insertion), STGF (semitendinosus gracilis free) and BPTB (bone-patellar tendon-bone)^19-21^.

Reference point 15cm above superior pole of patella was used to measure thigh circumference. Hormone levels (ooestrogen and progesterone) were done in the serum by chemiluminescence ADVIA Centaur XP system. Tunnel view at 45º of flexion and lateral view were used to calculate intercondylar notch and posterior tibial slope^22-23^. All patients underwent a standard post-operative rehabilitation protocol for six months. Rehabilitation protocol with closed-chain exercises were started from post-operative day 1, and open-chain exercises were introduced at three months of follow-up. During the first six weeks, patients were allowed unlimited range of motion and full weight bearing in a brace, and they performed straight leg raises and static quadriceps exercises. At six weeks, cycling was introduced in addition to the existing physiotherapy. At three months, light jogging was allowed. At six months, patients were allowed to practice sports and undergo endurance exercises for the next one to two months. Finally, after satisfactory performance by players in a practice game, patients were allowed to return to competitive sports. Because the study was a single-blind study, the surgical technique was not disclosed to the patient and the observer.

In statistical analysis, quantitative data was presented as mean ± SD or median and interquartile range, as appropriate. Normality of data was checked by Kolmogorov-Smirnov test of normality. For skewed data or scores, Mann-Whitney test for two groups was applied. For normally distributed data, two groups were compared using independent t-test. Proportions were compared using Chi-square or Fisher’s exact test, depending on their applicability. All the statistical tests were two-sided and performed at a significance level of 0.05. The analysis was conducted using IBM SPSS STATISTICS (version 22.0).

## Results

Out of 2,042 ACL reconstruction surgeries performed in the last 10 years, 452 (22.13%) met the inclusion criteria. A total of 64 patients suffered ACL re-injury and were included under Group I (26 patients on ipsilateral side and 38 patients on contralateral side). In Group II, out of remaining 388 sportspersons, 64 were randomly selected. Ipsilateral graft rupture rate was 5.7% and contralateral ACL injury was 8.4%. The average age of the patients was 24.90±4.06 years in Group I and 26.47±6.51 years in Group II. The mean follow-up of patients in Group I was 24.9±7.0 months, whereas the mean follow-up of Group II patients was 28.2±7.3 months. In Group I, two patients lost to follow-up and in Group II three patients lost to follow-up. The mean duration between injury and index surgery was 15.75±20.16 months in Group I and 18.11 ±19.9 months in Group II. The mean duration between index surgery and re-injury was 20.11 ±7.56 months. There were 4 (6.25%) females in Group 1 and 3 (4.68%) in Group II. In Group I, 36 patients sustained an injury to the right knee, whereas in Group II, 38 patients sustained an injury to the right knee. In both the groups’ non-contact mode of injury was more with 42 (65.62%) patients in Group I and 46 (71.87%) patients in Group II. Both the groups were comparable for body mass index; femoral tunnel length (graft length in tunnel was kept ≥ 15mm in all cases in both the groups), notch width index, type and size of graft; hormonal factors including oestrogen and progesterone and type of game played with p value >0.05 (Table I, Table II and Table III). Limb symmetry index (LSI) using single leg hop test was 90.2% in Group I, and 88.7% in Group II at a mean follow-up of six months. The difference between uninjured and injured limb using single leg hop test was not significant (p>0.05). In Group I, mean posterior tibial slope was 10.15±1.40º and in Group II mean posterior tibial slope was 8.53±2.20º with p value <0.05 (Table I). Arthrometric KT-1000 difference was found to be significant at one year follow-up with p value of <0.05 (Table I). Mean time to return to sports was found to be significant, with Group I having mean time to return to sports at 8.10±2.90 months and Group II having mean time to return to sports at 9.51±2.60 months (p<0.05) (Table I). In both the groups, difference between mean pre-injury and post-operative Tegner Activity Score at one year follow-up was found to be non-significant with p value of >0.05 (Table IV).

**Table I:** Comparison of continuous variables between two groups

	Group I Mean +/- S.D.	Group II Mean +/- S.D.	P value
Body Mass Index (n=64)	25.40±3.70	24.27±3.10	0.06
Thigh wasting at 12 months	2.50±1.17cm (n=62)	2.00±1.23cm (n=61)	0.02
KT Difference at 12 months (n=64)	2.90±0.60mm (n=62)	2.10±1.10mm (n=61)	0.00(<0.05)
Time to RTS (n=64)	8.10±2.90 months	9.51±2.60 months	0.00(<0.05)
Posterior Slope (n=64)	10.15±1.40°	8.53±2.20°	0.00(<0.05)
Notch width Index (n=64)	.28±.04	.29±.05	0.21
Size of Hamstring Graft (n=64)	7.40±1.13mm	7.54±1.10mm	0.47
Hormone levels (n=64)			
Oestrogen	32.87±19.40 pg/ml	35.19±18.07 pg/ml	0.48
Progesterone	1.70±1.30 ng/ml	1.4±.34 ng/ml	0.07
Femoral Tunnel Length (n=64)	34.05±5.91(mm)	35.34±6.04	0.22
Type of Graft(n=64)			
BPTB	16	17	0.92
STGPI	14	11	0.32
STGF	34	36	0.86

**Table II:** Comparison of type of game played between two groups

	Group I (n=64)	Group II (n=64)	p-value
Types of Game			0.845
Athletics	2	2	
Badminton	2	2	
Basketball	2	1	
Cricket	5	2	
Football	16	15	
Golf	0	1	
Gymnastics	1	0	
Handball	1	0	
Hockey	1	0	
Kabaddi	28	34	
Kho Kho	1	0	
Lawn tennis	0	1	
Martial arts	1	0	
Squash	0	1	
Volleyball	1	1	
Wrestling	4	3	

**Table III:** Graft type in Group I between ipsilateral and contralateral ACL injury

Type of Graft	Ipsilateral graft rupture (n=26)	Contralateral ACL injury (n=38)
BPTB	5	12
STGF	11	6
STGPI	10	20

## Discussion

The principle findings of this study was that mean time to return to sports of less than 9.5 months, mean thigh circumference difference of >2.5cm at one year follow-up, mean KT difference of >3mm at one year follow-up were statistically significant risk factors for ACL re-injury. Further, it was observed that posterior slope of >10º, was a statistically significant risk factor for causing ACL re-injury. Though, there was more graft rupture rate with hamstring free graft, we did not observed graft type to be a statistically significant risk factor with p value of >0.05.

It has already been reported in literature that quadriceps and hamstring weakness directly alter knee biomechanics and leads to increased anterior translation of tibia and thus make an athlete prone to ACL re-injury^24,25^. In addition to impaired activation of the involved-limb quadriceps, there is potential for activation failure in the uninvolved-limb quadriceps^26^. Whether it’s the atrophy of muscle or quadriceps activation failure which leads to weakness in strength of quadriceps is still controversial^26^. This necessitates the importance of thigh circumference measurement and arthrometric side to side measurement with help of KT-1000 at each follow-up to prevent re-injury to ACL.

Grindem *et al*^27^ reported that Return To Sports(RTS) should be delayed till nine months following primary ACLR. Similarly, Kegerreis *et al*^28^ and Sousa *et al*^29^ emphasised on the phasic manner over several months for progression to higher intensity exercises during rehabilitation to prevent further re-injury of ACL. However, Myer GD *et al*^30^ reported that ACL re-injury is independent of time frame for RTS. These contrasting studies in literature reiterate the need of further studies to better define a requisite timeframe for RTS at competitive level. This study helps in providing a possible answer to this controversy and reports a time frame of 9.5 months before an athlete can return back to competitive sports.

Kinematic evidence demonstrates that larger PTS results in increased anterior tibial translation over femur and hence increased strain on ACL^31^. Increased PTS as a risk factor for primary ACL injury has already been defined in literature conclusively but PTS as a cause of ACL re-injury is still controversial^30^. Webb *et al*^32^ reported augmented risk of ACL re-injury in patients with increased PTS. Contrasting to this, Stijak *et al*^33^ did not find any influence of PTS on ACL re-injury. This study helps in providing a possible answer and observed increased PTS as a risk factor for ACL re-injury.

Palmer *et al*^34^ observed that constitutional morphologic characteristics and anthropometric measurements considerably vary among different ethnic groups, which influence the NWI value. Alizadeh *et al*^35^ and Wolf *et al*^36^ reported no correlation of NWI with ACL injury while Al-Saeed *et al*^37^ reported, it’s the shape of notch rather than size of notch which is a risk factor for primary ACL injury. However, a meta-analysis reported that smaller NWI is a risk factor for primary ACL injury^38^. These contrasting results pushes for further need of literature to study this variable and this study provides a conclusive answer to this and reports notch width index is not a risk factor for ACL re-injury to either knee.

It is a known fact that, forces acting on knee joint at the time of injury are different in non-contact and contact injury. In non-contact injury, both rotational and bending forces are acting on the knee with proposed theories of impingement on the intercondylar notch, forceful quadriceps contraction, quadriceps-hamstring force imbalance and axial compressive forces while in the later, the main force is exerted due to bending forces^24,39-41^. Though previous literature, reports non-contact mode of injury as a risk factor for causing primary ACL injury, we in this study didn’t observed the same and hence report mode of injury is not a risk for causing ACL re-injury^42^.

In this study, the correlation of oestrogen and progesterone with ACL re-injury to either knee was found to be statistically non-significant. It is a known fact that, receptors for oestrogen and progesterone are present on ACL in both males and females^43,44^ wherein the former facilitates the collagen production and the latter inhibits the collagen production. Also, decreased fibroblast and pro-collagen production has been reported in tissue culture models when exposed to high concentrations of oestrogen^45,46^, emphasising that these hormones do play a significant role in tissue remodelling. Till now, no single case control or cohort study has been done in humans to conclusively define this variable as a possible risk factor. Though few case series or descriptive studies mentioned its role as a risk factor but still a consensus has not been reached^11^.

The limitations is this was a single-centre study with small sample size. Meniscal and chondral damage were not studied. The strengths of quadriceps (Q) and hamstring (H) and the Q/H could not be assessed individually. Thus we don’t have a clear understanding of flexor and extensor strength gap, as our study, relied on the thigh circumference difference as a way of defining the muscle weakness. Another, possible limitation of this study may be the use of radiographs which have a bigger error limit in calculating NWI, however, Gomes and Scarton^47^ concluded that, there is no appropriate and uniformly accepted reference in literature for the measurement of femoral inter-condylar notch. Further we propose large multicentric studies are required to further validate this study.

## Conclusion

Risk factors identified in the current study which contribute towards both ipsilateral graft rupture and contralateral ACL injury include posterior tibial slope of ≥ 10º, KT difference of ≥ 3.0mm at 12 months follow-up after primary ACLR, thigh atrophy of ≥ 2.50cm 12 months follow-up after primary ACLR, return to sports before 9.5 months after primary ACLR.
